# Influenza in Outpatient ILI Case-Patients in National Hospital-Based Surveillance, Bangladesh, 2007–2008

**DOI:** 10.1371/journal.pone.0008452

**Published:** 2009-12-29

**Authors:** Rashid Uz Zaman, A. S. M. Alamgir, Mustafizur Rahman, Eduardo Azziz-Baumgartner, Emily S. Gurley, M. Abu Yushuf Sharker, W. Abdullah Brooks, Tasnim Azim, Alicia M. Fry, Stephen Lindstrom, Larisa V. Gubareva, Xiyan Xu, Rebecca J. Garten, M. Jahangir Hossain, Salah Uddin Khan, Labib Imran Faruque, Syeda Shegufta Ameer, Alexander I. Klimov, Mahmudur Rahman, Stephen P. Luby

**Affiliations:** 1 International Centre for Diarrhoeal Disease Research, Bangladesh (ICDDR,B), Dhaka, Bangladesh; 2 Institute of Epidemiology, Disease Control, and Research (IEDCR), Dhaka, Bangladesh; 3 Centers for Disease Control and Prevention (CDC), Atlanta, Georgia, United States of America; 4 Johns Hopkins Bloomberg School of Public Health, Baltimore, Maryland, United States of America; University of Witwatersrand, South Africa

## Abstract

**Background:**

Recent population-based estimates in a Dhaka low-income community suggest that influenza was prevalent among children. To explore the epidemiology and seasonality of influenza throughout the country and among all age groups, we established nationally representative hospital-based surveillance necessary to guide influenza prevention and control efforts.

**Methodolgy/Principal Findings:**

We conducted influenza-like illness and severe acute respiratory illness sentinel surveillance in 12 hospitals across Bangladesh during May 2007–December 2008. We collected specimens from 3,699 patients, 385 (10%) which were influenza positive by real time RT-PCR. Among the sample-positive patients, 192 (51%) were type A and 188 (49%) were type B. Hemagglutinin subtyping of type A viruses detected 137 (71%) A/H1 and 55 (29%) A/H3, but no A/H5 or other novel influenza strains. The frequency of influenza cases was highest among children aged under 5 years (44%), while the proportions of laboratory confirmed cases was highest among participants aged 11–15 (18%). We applied kriging, a geo-statistical technique, to explore the spatial and temporal spread of influenza and found that, during 2008, influenza was first identified in large port cities and then gradually spread to other parts of the country. We identified a distinct influenza peak during the rainy season (May–September).

**Conclusions/Significance:**

Our surveillance data confirms that influenza is prevalent throughout Bangladesh, affecting a wide range of ages and causing considerable morbidity and hospital care. A unimodal influenza seasonality may allow Bangladesh to time annual influenza prevention messages and vaccination campaigns to reduce the national influenza burden. To scale-up such national interventions, we need to quantify the national rates of influenza and the economic burden associated with this disease through further studies.

## Introduction

Influenza is a major public health concern, annually infecting 5–15% of the global population, resulting in an estimated 250,000 to 500,000 deaths per year [1], [2]. In the United States the proportion of the population infected with influenza ranges between 5–20% resulting in an average of 36,000 annual deaths [3], [4]. The number of deaths in the United States related to these annual influenza epidemics during 1974–1994 was many times greater than the number of deaths caused by the 1957 and 1968 influenza pandemics [5]. The prevalence and burden of influenza are well described for the temperate countries in both the northern and southern hemispheres [4]–[13]. In those countries the seasonal peaks of influenza occur distinctly during the cold seasons [2], [12], [14]–[17]. Typically, elderly people and children aged under 5 years have the highest influenza morbidity and mortality and vaccination campaigns target these groups [2], [6], [7], [18].

In contrast to countries in temperate climates, much less is known about the epidemiology and seasonality of influenza in tropical countries. In recent years, there has been increasing data on the potential magnitude of influenza burden in sub-tropical and tropical areas. However these were predominantly sporadic outbreak reports or hospital-based studies from wealthier tropical countries [19], [20]. What has been lacking are data from surveillance in the tropics, although a few courtiers are notable exceptions. El Salvador, for example, reported repeated annual influenza epidemics during the rainy seasons [21]. Hospital surveillance in Kenya found 248 (38%) influenza positives out of 660 collected samples [22]. In Thailand the incidence was highest among the elderly over 55 years of age with epidemics occurring during June–September with an occasional increase of circulation during January and February. Thailand also quantified an annual influenza incidence ranging from 64–91 cases per 100,000 persons during 1993 and 2002 [23], [24]. Surveillance data from Pune and Chennai in India suggested that 5–12% of the influenza like illness (ILI) cases were due to influenza, especially during the rainy season [25]–[27].

Recent improvements in surveillance and laboratory capacity have allowed Bangladesh, a populous country with widespread outbreaks of H5N1 in poultry, to study the epidemiology and seasonality of human influenza and identify potentially novel strains, such as influenza A/(H5N1) and novel influenza A/(H1N1) [28], [29]. During 2004, the International Centre for Diarrhoeal Disease Research (ICDDR,B) established population based influenza surveillance in children younger than five years old in Kamalapur, a low income urban neighborhood in the capital city, Dhaka. After two years of surveillance investigators reported that 14% of children with acute respiratory infections had respiratory isolates that tested positive for influenza (84.5 episodes/1000 child/years). The surveillance suggested that influenza season occurred during April through September [30], [31]. This surveillance system also identified the one human case of infection with influenza A/(H5N1) in Bangladesh [32].

Based on the knowledge gained from the Kamalapur study, investigators from ICDDR,B, the Institute of Epidemiology, Disease Control and Research (IEDCR) of the Government of Bangladesh and Centers for Disease Control and Prevention (CDC), United States, collaborated to broaden influenza surveillance in this country. The primary objective was to understand the epidemiology and seasonality of influenza strains in Bangladesh from all areas and all age groups in the country. Aims included quantifying the prevalence of influenza infections among persons seeking care at the outpatient department of these hospitals, identifying circulating influenza virus strains, exploring seasonality, and characterizing clinical manifestation of influenza. In addition to these we also intended to identify novel influenza viruses among hospitalized case-patients. These data are important for public health decisions to prevent influenza in Bangladesh. Here we present the first 20 months of surveillance data from May 2007 to December 2008.

## Methods

### Ethics Statement

The Ethical Review Committee (ERC) of International Centre for Diarrhoeal Disease Research (ICDDR,B) reviewed and approved the protocol (#2007-002) on 22 March 2007. All the surveillance participants provided written informed consent during enrollment.

### Surveillance Sites and Personnel

**Figure 1:**
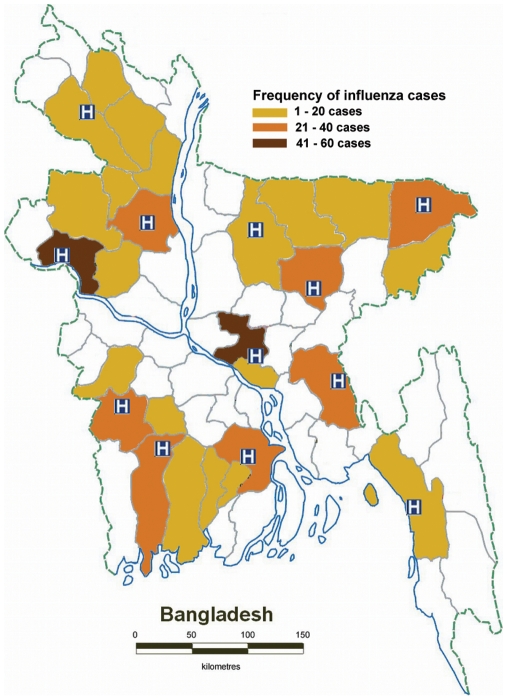
Global positioning system coordinated map of Bangladesh showing the surveillance sites and geographical distribution of influenza positive cases during May 2007–December 2008.

To establish a national hospital-based influenza surveillance system, we identified six government and six non-government hospitals located throughout Bangladesh in all six divisions (Figure 1). For selection criteria we considered population proportions of each six administrative divisions of the country, geographical location, public-private mixture, institutional capacity to carry out large scale surveillance and interest in participation. Among these surveillance sites ten are teaching hospitals, one is a district hospital and one is a faith-based hospital. Severe cases are frequently referred to these hospitals from the catchment areas. These hospitals treat between 200 and 1000 patients daily in their outpatient departments. Their inpatient capacity ranges between 250 and 900 beds with an average monthly bed occupancy rate of 100% to 200%.

We recruited a physician in each of the 12 sites from the hospitals' existing staff to oversee surveillance activities. ICDDR,B field assistants were deployed to help the surveillance physicians collect data and transport biological samples to the Virology Laboratory of ICDDR,B in Dhaka. The surveillance started in May 2007 and by October 2007 all 12 hospitals were enrolled in the surveillance network.

### Surveillance Methods

To determine the number of influenza positive ILI case-patients conducted active surveillance in outpatient departments affiliated with each hospital on two consecutive days each month. In addition, to identify novel influenza virus, we collected specimens from SARI case-patients from the hospitals' inpatient wards during those two days. After obtaining signed informed consent, the surveillance physicians collected throat and nasal swab from patients of all age groups visiting outpatient departments of those hospitals with influenza like illness (ILI), defined as subjective fever and (cough or sore throat). We also collected samples from the patients admitted in the medicine and pediatrics inpatient departments who met the case definition of severe acute respiratory illness (SARI), defined as fever (>38°C) and (cough or sore throat) and (shortness of breath or difficulty breathing). We excluded the children aged less than 5 years of age from inpatient SARI surveillance because childhood pneumonia is very common among this age group and samples from these cases, of which a large proportion may have been positive for other respiratory viruses, were expected to overwhelm our laboratory throughput. The surveillance physicians only collected specimens from those patients whose symptom onset was within seven days as virus can be more efficiently detected in respiratory specimens during the acute stage of infection. The surveillance physicians also performed physical examinations and recorded demographic and clinical information from the patients on a structured form. They collected data on demographics, potential work exposures for health care workers and poultry workers, travel history, clinical presentations, admission and discharge dates, symptoms, signs, provisional diagnosis, outcome of the admitted patients, available laboratory investigations, chest radiogram and treatment. The surveillance physicians collected specimens from up to 20 patients every month from each site. First from inpatient department the surveillance physicians collected samples from all eligible SARI case-patients. Then they moved to outpatient departments and collected samples until a total of 20 samples are collected. Immediately after collection, nasal and throat swabs were placed together in a single vial with viral transport media containing DMEM (Dullbecco's modified Eagle medium), 2.5% BSA (Bovine serum albumin) fraction V, 1% Glutamine, 2% HEPES (4-(2-hydroxyethyl)-1-piperazineethanesulfonic acid), 1% Penicillin-Streptomycin and Fungizone (250 µg/ml). The field assistants stored the specimens in refrigerators or cool boxes at 2–8°C in the field sites until transported.

Within 72 hours after collecting specimens, study personnel transported them in cool boxes to the Virology Laboratory of ICDDR,B. The specimens were aliquoted in a BSL-2 (bio-safety level - 2) safety cabinet and were stored in freezers at or below −70°C until analysis. For influenza testing, we performed real time reverse transcriptase polymerase chain reaction (rRT-PCR) [33]. Influenza A viruses were further subtyped with H1, H3 and H5 primers provided by Influenza Division at CDC. We strictly maintained the quality control of the laboratory testing. During rRT-PCR the ribonucleoprotein (RNP) was assessed to see whether the samples contain sufficient human cells. CDC periodically sent unknown samples to ICDDR,B laboratory and asked for the laboratory results for verification and all the test results were correct. We also periodically shipped randomly selected subsets of specimens to CDC for external verification, identification of unsubtypable influenza virus, nucleotide sequencing and anti-viral resistance testing using pyro-sequencing.

In addition to regular active surveillance, we also sought to identify clusters of severe acute respiratory illness defined as 3 or more patients aged >5 years admitted with severe acute respiratory illness, who live within a 30 minute walk (or within 3 kilometer radius) and who developed symptoms within 7 days of each other. To identify clusters of SARI, the surveillance physicians enlisted all the SARI cases, more than 5 years of age, in a registers throughout the month and looked for clusters based on above mentioned case-definition. We tested specimens of any hospitalized patient meeting the SARI case definition with history of exposure to a known or suspected H5N1 outbreak in poultry on priority basis and collected acute and convalescent serum for serologic testing at CDC.

### Sample Size and Data Analysis

We assumed that an influenza virus was present in at least 1% of the population that has influenza-like-illness. An annual sample of 1,519 was estimated to detect the proportion±0.5% with 95% level of confidence and with >80% power. We collected ∼20 ILI samples from each hospital each month and finally over 20 months we collected 3621 samples, i.e. 2,172 samples per year, which was greater than the estimated sample size. We did not considered inpatient SARI cases during sample size calculations since the SARI surveillance was designed to identify novel virus and clusters of severe respiratory illness cases. Therefore the SARI component of the surveillance was not representative.

We collected weather data for each surveillance district from Bangladesh meteorological department and explored association of temperature, relative humidity, average sunlight and rainfall with the proportion of laboratory confirmed influenza. We also collected data from Department of Livestock of Government of Bangladesh to compare the seasonality of poultry outbreaks with influenza A/(H5N1) and human seasonal influenza.

We analyzed the data using SPSS and STATA and performed correlation analysis, two-way contingency tables with chi-square test or Fisher's exact test for association, chi-square test for trend and logistic regression to study the association of proportion of laboratory confirmed influenza with different variables. We applied kriging, a geo-statistical technique, to determine influenza appearance in each surveillance area and to explore the spatiotemporal spreading pattern of influenza virus over Bangladesh. Statistical software R (Language for computing and graphics developed at Bell Laboratories) was used for kriging.

## Results

### Demographics

Between May 2007 and December 2008, we collected specimens from 3,699 case-patients. Among them, 3,621 (98%) were outpatient ILI case-patients and 78 (2%) were inpatient SARI case-patients. The mean age of ILI case-patients was 15 (SE±0.28) years ranging from less than 1 month to 90 years. For SARI case-patient it was 40 (SE±2.23) years ranging from 7–85 years. Among the ILI case-patients 1,624 (45%) and among SARI case-patients 11 (14%) were female.

### Proportion of Case Patients with rRT-PCR Confirmed Influenza

**Figure 2:**
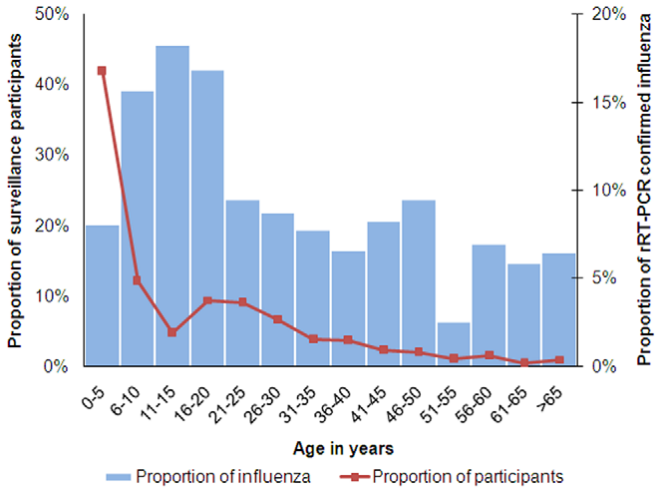
Proportion with rRTPCR confirmed influenza among ILI case-patients in different age groups.

Among ILI case-patients 375 (10%) and among SARI case-patients 5 (6%) tested positive for influenza (Table 1). The mean age of influenza positive ILI case-patients was 14 (SE±0.73) years, ranging from 2 months to 70 years. For SARI case-patients it was 30 (SE±5.36) ranged between 21 and 50 years. Among ILI case-patients children under 5 years of age constituted 42% of the total cases, while the proportion of laboratory-confirmed influenza cases were higher among participants aged 6 to 20 years (Figure 2). Among outpatient ILI 1,997 male case-patients 224 (11%) had influenza in comparison to 151 (9%) of 1,624 females (p-value = 0.06). Among 78 hospitalized SARI case-patients 62 (86%) were male and all five case-patient who tested positive with influenza belonged to this sex. During the surveillance period influenza positive samples were found from all parts of Bangladesh under surveillance (Figure 1). During October 2007 to September 2008, when surveillance was operational in all 12 hospitals, the percent of ILI samples that tested positive for influenza were 9% in Dhaka, Mymensingh, Chittagong, and Jessore, 10% in Dinajpur, Comilla and Khulna, 11% in Rajshahi, 12% in Kishoregonj and Barisal and 13% in Bogra and Sylhet. Among 3,621 ILI case-patients, we collected 1934 (53%) samples from public and 1,687 (47%) samples from the private hospitals. Among samples from public hospitals 203 (10%) were influenza positive, in comparison to 172 (10%) from private facilities, indicating no significant differences (p-value = 0.8). Among 78 hospitalized SARI cases 54 (69%) were from public and 24 (31%) were from private facilities. All five cases that tested positive with influenza were from public facilities.

**Table 1:** Proportion of ILI and SARI case patients with influenza virus infection.

	ILI, no. (%)	SARI, no. (%)	Total, no. (%)
	n = 3621	n = 78	n = 3699
Influenza positives	375 (10)	5 (6)	380 (10)
Types	n = 375	n = 5	n = 380
Influenza A	188 (50)	4 (80)	192 (51)
Influenza B	187 (50)	1 (20)	188 (49)
Subtypes	n = 188	n = 4	n = 192
Influenza A/H1	137 (73)	0 (0)	137 (71)
Influenza A/H3	51 (27)	4 (100)	55 (29)
Influenza A/H5	0 (0)	0 (0)	0 (0)

### Circulating Strains

**Figure 3:**
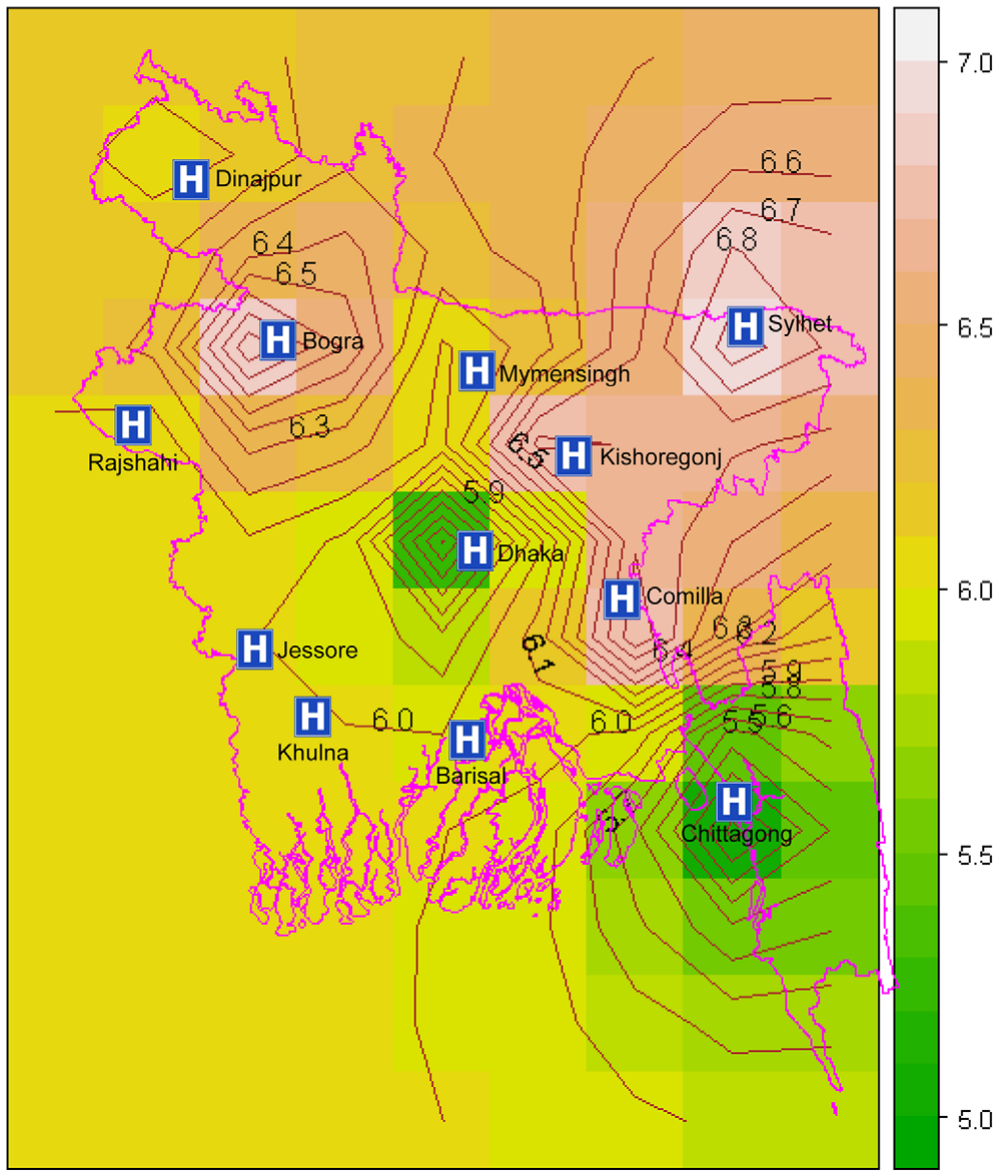
Kriging prediction of month of onset of influenza season in different parts of Bangladesh. We divided the total study area into 5 km by 5 km regions. Then we estimated the month of first appearance of influenza for 2008 in each region based on the distance weighted first positivity month of the surrounding locations using interpolation. We plotted the estimated values using a color scale. We used the observed months as integers. We also include contour lines in the plot to specify the areas where the interpolated values were the same. In this figure 5 = May, 6 = June and 7 = July and the intermediate values are the mid-months.

While both influenza A and B virus infections were equally present among ILI cases, influenza A/H3 was predominant among SARI cases. Both strains of seasonal influenza A and influenza B were detected, while none of the cases from inpatients or outpatients were influenza A/H5 or novel strains or unsubtypable (Table 1). In Chittagong, in the south-east part of Bangladesh, influenza A was more common, whereas in Sylhet, in the north-east part, influenza B was more common. Kriging analyses of the 2008 influenza season suggest that surveillance samples tested positive during May (month 5) in Chittagong (i.e. a large maritime port city)and Dhaka (i.e. the capital and largest city), before gradually spreading to the other parts of Bangladesh (Figure 3).

There was wide variation in the pattern of resistance to anti-viral drugs among 61 randomly selected specimens sent to CDC for anti-viral resistance testing. The influenza A/(H1N1) and influenza A/(H3N2) viruses circulated in 2007 were sensitive to the neuraminidase inhibitors oseltamivir and zanamivir, while most were resistant to the adamantane group of drugs, amantadine and rimantadine. In 2008, all the influenza A/(H1N1) viruses were resistant to oseltamivir and all the A/(H3N2) viruses were resistant to adamantanes.

### Seasonality

**Figure 4:**
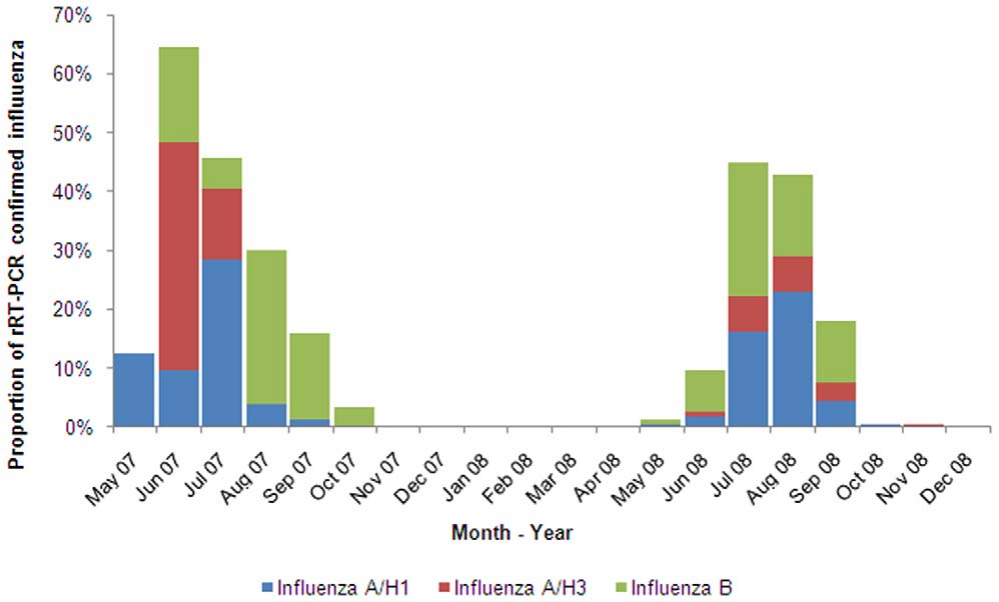
Seasonality of influenza in Bangladesh.

**Figure 5:**
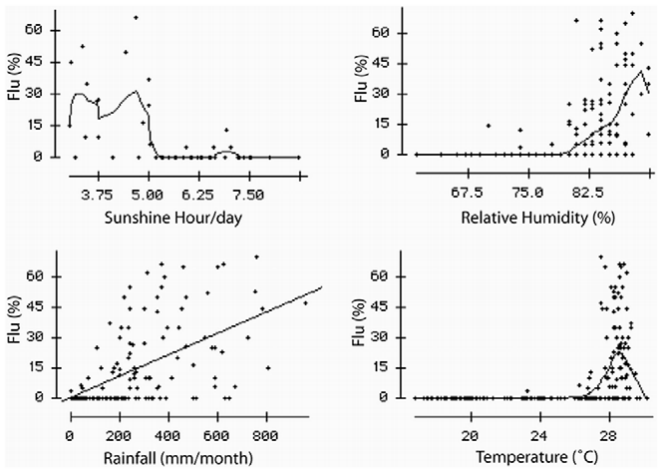
Correlation of percent positivity of influenza with the average monthly rainfall, average temperature, average relative humidity, and average sunlight hours.

**Figure 6:**
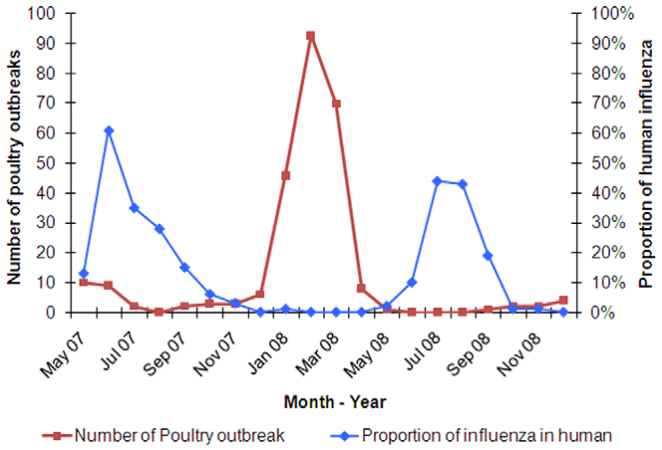
Non-coinciding seasonality of seasonal influenza in human and avian influenza in poultry in Bangladesh.

The influenza activity was seasonal, unimodal and sharply demarcated (Figure 4). The percentage of positive samples exceeded 10% during June to September when most (96%) influenza positive specimens were collected. During the month of peak activity, 45–62% of the ILI case-patients tested positive for influenza. In contrast, we identified very few influenza infections during October–April. During 2007 influenza type A infections were identified before type B while during 2008, type A and B activity occurred concurrently. The unimodal peak was concurrent with the rainy season in Bangladesh. Higher percent positivity appears concurrent with months during which there were low hours of sunlight and elevated temperatures, relative humidity, and rainfall (Figure 5). The seasonality of reported outbreaks of H5N1 in poultry in Bangladesh does not coincide with the seasonality of human influenza (Figure 6).

### Clinical Presentations

Influenza confirmed case-patients presented with classic clinical signs and symptoms of disease. Fever was essential criterion in the case-definitions and so was present in all ILI and SARI case-patients. In addition they frequently presented with cough and runny nose (Table 2).

**Table 2:** Clinical presentations in influenza and non-influenza case-patients.

Symptoms	Influenza positive ILI no. (%) n = 375	Influenza negative ILI no. (%) n = 3246	Influenza positive SARI no. (%) n = 5	Influenza negative SARI no. (%) n = 73
Fever*	375 (100)	3246 (100)	5 (100)	73 (100)
Cough	354 (94)	3058 (94)	5 (100)	72 (99)
Sore throat	78 (21)	692 (21)	0 (0)	10 (14)
Difficulty breathing	56 (15)	876 (27)**	5 (100)	5 (100)
Runny nose	290 (77)	2218 (68)**	3 (60)	17 (23)
Headache	127 (34)	785 (24)**	3 (60)	39 (53)
Diarrhea	15 (4)	217 (7)	0 (0)	3 (4)

* Fever was the essential criterion for enrolment.

** Significant.

Among the case-patients with influenza infection, 41% had documented temperature greater than 38°C during sample collection. The mean temperature was 37.9 (SE±0.08)°C ranging between 35.6°C and 40.3°C. The mean duration of fever was 3 (SE±0.08) days at the time of sample collection. We found age-specific tachypnea in 15% of 2–11 months children (>50/min), 13% of 12–59 months children (>40/min), 39% of 5–9 years children (>24/min), 60% of 10–14 years children (>24/min) and 46% of participants aged 15 years and above (>20/min). On auscultation 11% had rhonchi and 14% had crepitations. The mental status was normal in 79% of case-patients, while the remaining 21% were irritable, less active or lethargic. No case-patient had cyanosis or was unconscious during the time of sample collection. Hematological mean counts of laboratory-confirmed influenza cases were leukocytes 8033 (SE±2092)/cu mm, neutrophil 42% (SE±7), lymphocyte 46% (SE±5.6) and erythrocyte sedimentation rate 17 (SE±9) mm after first hour.

### Treatment Pattern

Typically, influenza positive case patients were managed with supportive care and antibiotics. Out of 3621 ILI case-patients, 3240 (89%) were prescribed medications, 17 (1%) were referred to other hospitals, 49 (2%) were advised for admission and 212 (7%) were referred for laboratory investigations outside of the hospital. Treatment information was not available in 103 (3%) case-patients where samples were obtained before prescribing medicines. Among 3240 ILI case-patients who received treatment, 2741 (85%) received antibiotics. Among 78 inpatient SARI case-patients 77 (99%) received antibiotics. While all (100%) of the 47 SARI case-patients with abnormal chest radiogram were treated with antibiotics. Analysis showed that there was an increasing linear trend between prescribing antibiotics and the severity of disease (p-value<0.001). Two (<1%) of 3240 prescribed ILI case-patients received antivirals; one was given Oseltamivir and the other Acyclovir. While six (8%) of the 78 SARI case-patients received antiviral drugs; two of them received Oseltamivir, two received Acyclovir and two received Adefovir. This suggests that the SARI case-patients were more likely to received antivirals than the ILI case-patients (p-value<0.001).

### Health Care Workers, Poultry Exposures, and Suspected H5 Cases

Hospital and event surveillance did not identify any clusters of severe acute respiratory illness or novel strains of influenza. Thirty-seven percent (1280/3486) of patients reported raising poultry in their homes including 9% (115/1280) of people with confirmed influenza. We collected specimens from 60 health care workers and 37 poultry workers during surveillance. Among them, 15% of health care workers and 6% of poultry workers were found positive for influenza virus, but none with influenza A/H5. We identified two suspected human influenza A/H5 cases, both tested negative in rRT-PCR and serologically.

## Discussion

This surveillance data confirms that influenza is prevalent throughout Bangladesh, affects all age groups, and causes considerable morbidity. These data are in agreement with recently published papers from El Salvador, Kenya, Thailand and India that also demonstrated prevalent seasonal influenza epidemics in the tropics [21], [22], [24]–[26]. Our findings strengthen the data highlighting seasonal influenza as a global contributor to respiratory disease burden and it is important to include tropical countries in global influenza prevention activities. The unimodal and distinct seasonality of human influenza in Bangladesh provides an opportunity to explore measures to prevent influenza by non-pharmaceutical interventions, such as annual handwashing campaign, respiratory hygiene campaigns and pharmaceutical interventions, such as vaccination, which is not yet introduced in Bangladesh.

Acute respiratory illness (ARI) contributes to 21% of deaths of children aged less than 5 years in Bangladesh and contributes largely to the 31% deaths due to possible serious infections in this country [34]. We found that all age groups were affected with influenza in Bangladesh. Nearly half of the case-patients were less than 5 years old, which suggest high rates of ARI. The proportion of persons with symptoms who have rRT-PCR confirmed influenza is greater among toddlers and teen-agers, and lower in the youngest children and oldest adults, where influenza causes illness severe enough to seek hospital visits or admissions. Higher proportions of influenza among school-aged children suggest that school-based non-pharmaceutical interventions or vaccination may be worth considering for preventing influenza in Bangladesh. Causing over 40% of acute respiratory illness in its peak, influenza could be an important cause of ARI in Bangladesh. Preventing influenza could contribute to mortality reduction in children under five and achieving the millennium development goal of reducing infant and childhood mortality (MDG-4) [35].

Influenza virus subtypes found in Bangladesh were similar to viruses that circulated around the globe during 2007-08 [1], [2]. Even though our inpatient SARI samples were few, results suggest that influenza A/H3 virus caused more severe illness requiring hospitalizations. This higher virulence of influenza A/H3 over influenza A/H1 and influenza B is well established in studies from temperate regions [2], [5], [36]–[39]. Kriging analyses suggested that in May 2008, the influenza possibly first introduced in Chittagong and Dhaka and then gradually to the north-west of Bangladesh. Influenza may be introduced to Chittagong or Dhaka through the air ports in both cities or maritime port in Chittagong or it may remain at low levels of circulation throughout the year and then flare up during the appropriate environmental conditions [36]. However our surveillance did not found any evidence of year round circulation. In terms of spatial spread, it is possible that influenza spread rapidly between Chittagong and Dhaka because of the frequent travel between these two densely populated cities. These patterns of outward spread from dense population centers appear similar to the US, Brazil, and Japan [40]–[43]. We ran the kriging analysis with data from the 2008 influenza season only. We expect to have more information on this with subsequent years of surveillance.

Surveillance data suggest that May–September was the peak influenza season in Bangladesh which is offset from the influenza A (H5N1) season in poultry. The seasonality is consistent with reported seasonality of influenza infection from the population-based surveillance in the Kamalapur neighborhood of Dhaka city, where the peak season was April to September during 2004–2006 [30]. This time of the year is typically considered the rainy season in Bangladesh and during these two seasons of surveillance these months had higher rainfall as evident in the weather data from Bangladesh meteorological department. We found influenza positivity was concurrent with increased rainfall, temperature, and relative humidity consistent with recently published papers on influenza and climate [44], [45]. It is possible that, during the monsoons, people spend more time indoors in small poorly ventilated spaces which may increase influenza transmission. In contrast, the influenza A (H5N1) season in poultry occurs during October–March which is the time when wild birds migrate through Bangladesh [46].

Our surveillance system did not identify human infection with influenza A/H5 or other novel influenza strains in Bangladesh. Although a significant proportion of Bangladeshis do not routinely seek medical care for respiratory illness [34], [47], the findings from this nationwide surveillance suggest that human infections with H5 or other novel influenza viruses were not commonly occurring during the study period. The seasonality of human seasonal influenza does not coincide with the seasonality of H5N1 influenza in poultry, which might reduce opportunities for reassortment of avian strain with a human strain in Bangladesh [48], [49].

This surveillance has some important limitations. Our surveillance does not estimate the incidence and prevalence of influenza and so provides limited information on the burden of disease in the population. Duration of surveillance is also a limitation. This paper covered 20 months of surveillance data. We will be able to comment more robustly on the epidemiology and seasonality of influenza in Bangladesh after gathering a few more years of surveillance data. We conducted the surveillance in two consecutive days in each month; therefore we may have missed the peak influenza activity in some places. Another limitation is enrolment of a fewer number of SARI case-patients from inpatient departments than anticipated. We collected samples from the SARI case-patients who were admitted during two days of surveillance in each month. Moreover many SARI case-patients got admitted to the hospital after seven days of symptom onset. Broadening the SARI case definition by not including difficulty breathing or shortness of breath would increase the sensitivity of the case definition. Since the inpatient SARI surveillance was designed with the intent to identify novel strains of influenza in the context of limited laboratory capacity, we did not enrolled children less than 5 years of age for this component of the surveillance. In May 2009 we amended the surveillance protocol and started obtaining comprehensive epidemiologic information about this important age group that is at high risk of complications from influenza disease and sampling hospitalized children with severe pneumonia aged less than 5 years. Another limitation was that our case definition for clusters was very specific. Identifying three or more cases from the same locality within 7 days of symptom onset was probably appropriate for early detection of large outbreaks, but not sensitive enough to capture small outbreaks.

Findings from this surveillance confirm that influenza is common among all age groups throughout Bangladesh. Human influenza epidemics mostly occur during the rainy season in Bangladesh which is during a different time of the year than the poultry influenza A (H5N1) avian influenza season. The identification of seasonality in human influenza activity which is different from that of H5N1 activity in poultry provides public health agencies with an opportunity to time annual prevention strategies and conduct surveillance for unusual ILI or SARI outbreaks out of season. Continued and enhanced hospital surveillance focusing on severe hospitalized influenza cases is important to characterize severe disease and identify novel respiratory viruses. Studies on the epidemiology and financial burden of influenza are needed to determine the public health and economic burden of this disease and to guide public health interventions intended to reduce influenza infection in Bangladesh.
